# Aging-Associated Changes to Intrinsic Neuronal Excitability in the Bed Nucleus of the Stria Terminalis Is Cell Type-Dependent

**DOI:** 10.3389/fnagi.2017.00424

**Published:** 2017-12-22

**Authors:** Hannah E. Smithers, John R. Terry, Jon T. Brown, Andrew D. Randall

**Affiliations:** ^1^Hatherly Laboratory, Institute of Biomedical and Clinical Science, University of Exeter Medical School, Exeter, United Kingdom; ^2^College of Engineering, Mathematics and Physical Sciences, Living Systems Institute, University of Exeter, Exeter, United Kingdom

**Keywords:** bed nucleus of the stria terminalis (BNST), aging, female, electrophysiology, whole cell patch clamp, hyperexcitability

## Abstract

Intrinsic neuronal excitability has been reported to change during normal aging. The bed nucleus of the stria terminalis (BNST), a limbic forebrain structure, is involved in fear, stress and anxiety; behavioral features that exhibit age-dependent properties. To examine the effect of aging on intrinsic neuronal properties in BNST we compared patch clamp recordings from cohorts of female mice at two ages, 3–4 months (Young) and 29–30 months (Aged) focusing on 2 types of BNST neurons. Aged Type I neurons exhibited a hyperpolarized resting membrane potential (RMP) of circa -80 mV compared to circa -70 mV in the Young. A key finding in this study is a hyper-excitability of Type II neurons with age reflected in an increase in firing frequency in response to depolarizing current injections; activation of Type II neurons is believed to dampen anxiety like responses. Such age-related changes in intrinsic neurophysiological function are likely to modulate how the limbic system, acting via BNST, shapes function in the HPA-axis.

## Introduction

Chronic stress, anxiety and related mood disorders have a substantial effect on the well-being of all global societies. In the developed world, conditions within this spectrum of disorders have become a major economic burden, in terms of both direct healthcare costs and lost economic productivity (Kessler et al., 2001).

Although seen at most ages from childhood onward, the development and nature of chronic stress exhibits a complex age-related nature (Garrido, 2011; Prenderville et al., 2015) the basis of which is not fully understood. Many physiological manifestations of stress-related disorders are ultimately affected via the hypothalamus. This includes the classical neuroendocrine hypothalamo-pituitary-adrenocortical (HPA) axis, alongside a range of other neural circuits for example hypothalamic control of various brainstem nuclei that mediate peripheral homeostatic responses such as blood pressure regulation. Stress-eliciting neural drive, however, is thought typically to have it genesis in higher centers, in particular the circuits of the limbic system which are known to integrate multiple complex factors to shape emotion and mood.

Although the mammalian limbic system unquestionably has pivotal roles in eliciting and modulating stress signaling, it is not extensively connected to key hypothalamic areas involved in stress responses, including the paraventricular hypothalamic nucleus (PVN). The PVN is pivotal in this regard as it releases corticotrophin-releasing factor (CRF) into the median eminence to drive pituitary adrenocorticotropic hormone (ACTH) release into the systemic circulation and ultimately glucocorticoid release from the adrenal cortex. The PVN also exerts substantial influences on autonomic activity via its connections to various brainstem/spinal cord networks (Kc and Dick, 2010).

Due to the above mentioned lack of direct connectivity, limbic system influences on hypothalamic and brain stem-dependent outcomes are necessarily relayed through intermediate brain areas. One area of particular interest in this regard is the bed nucleus of the stria terminalis (BNST). In the mammalian brain the BNST is located in the caudal pallidum, adjacent to the caudate putamen, and either side of the anterior commissure. It receives afferent projects from many areas of the limbic system including the amygdala, the ventral subiculum, the hippocampus and the prefrontal cortex. Recent brain imaging-based analysis of BNST connectivity in humans indicates a very similar connectivity to that described in rodents, indicating that functional studies in rodents are likely to reflect largely human neural physiology. Furthermore, multiple functional magnetic resonance imaging (fMRI) studies in man have demonstrated the role of BNST in the human central nervous systems (CNS) response to fear and anxious anticipation (Straube et al., 2007; Somerville et al., 2010; Yassa et al., 2012).

The BNST comprises various different subregions each with highly specific connectivity to other brain regions. The anterolateral area has extensive connections with the hypothalamus, midbrain and lower brainstem regions. These brain regions are associated with pain, emotional processing, reward and autonomic function (Daniel and Rainnie, 2016). This area is also one of the most characterized regions of the BNST with regards to whole cell patch clamp electrophysiology. For these reasons this study focused on the antereolateral area (BNST^ALG^) (Hammack et al., 2007).

There are three types of cells located in the BNST^ALG^ which can be characterized based on their electrophysiological properties (Hammack et al., 2007). A recent review by Daniel and Rainnie (2016) has hypothesized the role that each of these cell types play in downstream physiological effects. CRF cells which also co-express glutamate are primarily located in the Oval component of the dorsal area of the anterolateral BNST and the fusiform nucleus in the medial part of the anterolateral area. The physiological properties of these CRF cells correlate with type III neurons, also 95% of type III neurons express CRF mRNA (Dabrowska et al., 2013). As selective activation of CRF cells in the oval BNST nucleus leads to increased anxiety behaviors (Sahuque et al., 2006) this population is believed to represents an ‘anxiety on’ switch. In an unpublished observation from the Rainnie lab (Daniel and Rainnie, 2016) it was observed that only type II neurons express mRNA for protein kinase C-δ (PKC-δ). In the central amygdala (CeA) the PKC-δ^+^ neurons represent an ‘anxiety off’ switch through reciprocal inhibition of CRF positive cells. Given the similarities between the amygdala and the BNST this would translate to a situation in which type III neurons represent anxiety on and type II neurons represent anxiety off switch (Daniel and Rainnie, 2016). The role of Type I neurons have not yet been examined in depth; Daniel and Rainnie (2016) have hypothesized an anxiety off role for this population. However, in unpublished observation from our lab we have shown that heightened excitability of Type I neurons correlated with an increase in anxiety like response on the elevated zero maze which could indicate an anxiety inducing role.

We hypothesized that known changes to HPA activity associated with healthy aging (Ferrari et al., 2001; Dalm et al., 2005) may, at least in part, reflect alterations in key circuits that modulate the activity of hypothalamic CRF releasing neurons. Given the direct upstream connectivity of the BNST to the paraventricular nucleus, and the resulting influence on this structure’s functionality, we performed a study in which we compared the neurophysiological properties of Young (3–4 month old) and Aged (29–30 month old) neurons in the dorsal part of the anterolateral area of the BNST. To our knowledge this is the first cell-level study to examine the effects of healthy aging on the neurophysiological function of the BNST.

## Materials and Methods

### Animals

All experiments were carried out in accordance with the animals (scientific procedures) act 1986. All tissues for this study were harvested from female C57-Bl6 mice purchased from Charles River. For their entire lifespan animals had *ad libitum* access to both food and water and were housed on a 12/12 light-dark cycle. In this investigation animals aged 3–4 months (Young) were compared to animals aged 29–30 months (Aged). Experimental days employing brain slices obtained from the two different ages of mouse were interleaved throughout the duration of the study.

### Slice Preparation

Animals were killed by cervical dislocation in accordance with schedule 1 of the UK Animals (Scientific Procedures) Act 1986. The skull was opened and the brain was rapidly removed and placed immediately in an ice-cold slicing medium consisting of (in mM): 189 Sucrose, 10 D-Glucose, 26 NaHCO_3_, 3 KCl, 5 MgSO_4_, 0.1 CaCl_2_, 1.25 NaH_2_PO_4_. A Leica VT1200 vibratome was then used to cut serial 300 μm thick coronal sections. Following their preparation slices were allowed to recover at room temperature for at least 60 min in our standard artificial cerebrospinal fluid (aCSF). This was composed of (in mM):124 NaCl, 3 KCl, 24 NaHCO_3_, 1.25 NaH_2_PO_4_, 2 CaCl_2_, 1 MgSO_4_, 10 D-Glucose, and was continuously gassed with carbogen (i.e., 95%O_2_,5%CO_2_)

Slices containing the BNST^ALG^ came from approximately Bregma -0.1 to +0.3, and were identified with the aid of the Paxinos and Franklin mouse brain atlas using the anterior commissure as a key landmark. Recordings were carried out in the dorsal portion of the BNST^ALG^. Typically one or two suitable BNST-containing coronal sections per animal could be used and by bisecting these along the dorsal-ventral midline we were able to obtain two to four usable tissue sections per mouse.

### Electrophysiological Recordings

All recordings were made using the whole cell patch clamp technique. The BNST containing brain slice was transferred into a submerged recording chamber which was perfused with gassed aCSF and maintained at a temperature of ∼34.5∘C. The recording chamber was mounted on the stage of an upright microscope (Olympus BX51).

A Flaming Browning P-97 micropipette puller was used to produce the microelectrodes used in this study. These had a resistance of 3–5 MΩ when filled with the K-Gluconate-based internal solution used for all recordings. This was composed of (in mM): 130 K-Gluconate, 20 KCl, 10 HEPES free acid, 0.2 EGTA, 0.3 GTP-Na salt, ATP-Mg salt, pH adjusted to 7.3 with KOH. The 15 mV junction potential error produced by pairing this pipette solution with our aCSF was corrected for during analysis.

Cells within the BNST were visually identified using the microscope’s infrared differential interference contrast optics and a coupled IR-sensitive CMOS camera (Thor Labs). All recordings were made with a Multiclamp 700B amplifier (Molecular Devices) interfaced to a Digidata 1440A (Molecular Devices). Experiments were controlled and data collected using the Clampex program within the pClamp 10.4 software suite. All data were stored directly onto a personal computer (Hewlett-Packard) and backed-up to a network drive.

### Electrophysiological Protocols

A sequential series of protocols were carried out in both voltage clamp and current clamp mode to assess the intrinsic and synaptic properties of BNST neurons. Initially cells were voltage clamped at a holding potential of -70 mV for 60 s. Here intermittent spontaneous postsynaptic currents could be observed. We recorded the average holding current, and following their detection, the mean frequency and amplitude of the spontaneous inward-going synaptic events were determined. Following the initial 60 s period in voltage clamp, the amplifier was switched to current clamp mode for the remainder of the recording allowing cellular voltage responses to be studied. Firstly, we recorded a period of activity in the absence of any injected current (i.e., at the resting potential) this allowed us to assess both the resting potential and also the proportion of cells exhibiting any spontaneous action potential firing.

Next by the application of a suitable level of bias current cells were set at a prestimulus membrane potential of -70 or -80 mV. Once the prestimulus membrane potential was set to the desired level a series of nine, 500 ms duration, current injections ranging from -40 to +80 pA in 15 pA increments was applied to each cell; the time between the stimuli was 10 s. The hyperpolarization caused by the first, i.e., -40 pA, current injection was used to determine passive, subthreshold membrane properties. Input resistance was calculated from the difference between the pre-stimulus voltage and average voltage during the final 100 ms of the hyperpolarizing current stimulus. The series of depolarizing pulses were used to characterize the firing properties of the cells. The action potential properties of each cell were characterized from the first action potential generated in response to the depolarizing steps. For each recording we measured action potential threshold, maximum rate of rise, zenith (i.e., peak depolarization) and width. Another way to examine excitability is to look at rheobase (i.e., the amount of depolarizing current required to elicit any spiking). This was achieved by applying current injections lasting 300 ms that were increased in amplitude by 2 pA per sweep until a spike was observed, the time between stimuli was 1 s. Cells were sorted into 3 different populations based on the criteria outlined by Hammack et al. (2007).

### Data Analysis

Data were analyzed using a range of custom written MATLAB scripts and pClamp 10.4 software. Determination of appropriate statistical test was based on assessment of normal distribution using the Shapiro–Wilk normality test. A number of properties were examined from only one membrane potential, i.e., firing frequency at rest; for these parameters if data were normally distributed an unpaired *t*-test was carried out, if data were not normally distributed a Mann–Whitney U was carried out. Part of the data was examined from two prestimulus potentials; if the data was normally distributed at both prestimulus membrane potentials a repeated measure two-way ANOVA was carried out with age as the between subject effect and prestimulus membrane potential as the within subject effect. If the data were not normally distributed a Mann–Whitney *U* test was performed at each prestimulus membrane potential. If the data were normally distributed at only one prestimulus membrane potential an unpaired *t*-test was carried out on this set and a Mann–Whitney U on the non-normally distributed set. Firing frequencies in response to each depolarizing current injection were analyzed via repeated measure three-way ANOVA. All statistics were carried out in SPSS. Proportion of cells firing was determined by chi squared, these tests were carried out in Excel. Figures were prepared with Origin 2016.

This study was exempt from approval by an ethics committee; animals used in this study were killed by cervical dislocation, this procedure is regulated by the schedule 1 of the animals (scientific procedures) act of 1986 of the United Kingdom.

## Results

A total of 28 cells from the Aged cohort and 43 cells from the Young cohort were recorded in the BNST^ALG^. The aged neurons consisted of 8 Type I cells, 16 Type II cells and 4 Type III cells, the young neurons consisted of 18 Type I cells, 18 Type II cells and 7 Type III neurons. Due to the low numbers of Type III cells recorded we did not make cross-age comparisons and they are not included in this report.

### Type I Neurons

**FIGURE 1:**
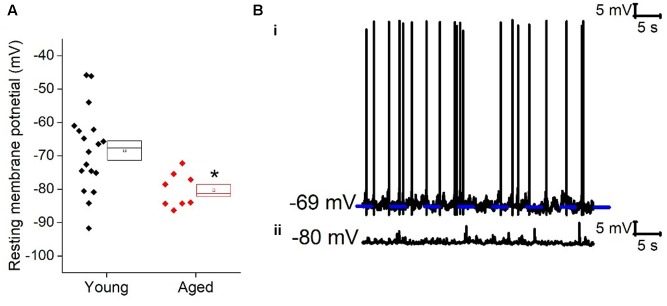
Aged Type I neurons have a hyperpolarized resting membrane potential. **(A)** Resting membrane potential (RMP), **(B)** Sample cells at resting potential either firing spontaneous action potentials (i) or not firing at rest (ii).

Cells were placed into current clamp to examine properties at their resting membrane potential; the mean resting membrane potential of the young cohort (-68 ± 3 mV, *n* = 18) was over 10 mV more depolarized than that of the Aged population (-80 ± 2 mV, *n* = 8), (unpaired *t*-test, *p* = 0.01, *t* = 2.6, **Figure 1**). The proportion of cells exhibiting any spontaneous action potential firing was 12 % in the Aged slices (1 of 8 cells) while 50% were firing in the Young group (9 of 18 cells; Chi-squared test *p* = 0.07). Of the cells which did not fire spontaneously there was no difference in rheobase (Young: 46 ± 11 pA, *n* = 5, Aged: 34 ± 11 pA, *n* = 7, Mann–Whitney U, *p* = 0.3, *Z* = -1).

Holding cells at a set membrane potential was achieved by application of suitable level of “steady state” bias current. The amount of current required to hold cells at -70 mV (Young: 5.5 ± 6 pA, *n* = 18, Aged: 23.6 ± 5 pA, *n* = 7) and -80 mV (Young: -27 ± 7 pA, *n* = 19, Aged: 4 ± 3 pA, *n* = 7) was dependent on age (repeated measure two way ANOVA, age *p* = 0.03, *F* = 5), reflecting the age-dependence of resting membrane potential.

**FIGURE 2:**
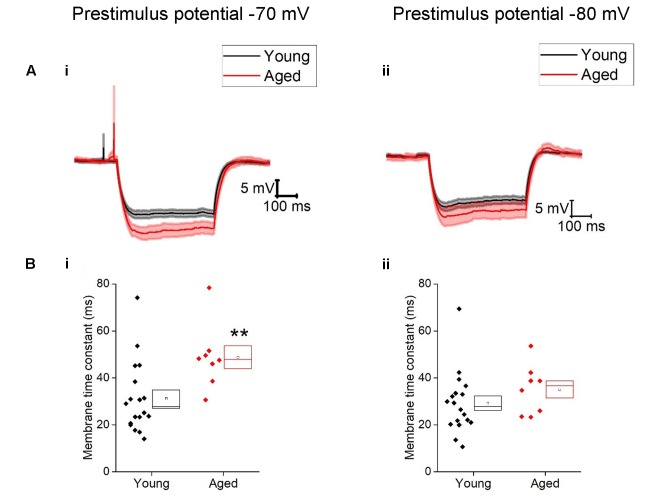
Passive membrane properties of type I cells. **(A)** Averaged traces of voltage response to a -40 pA current step in the Young and Aged cohort from a prestimulus membrane potential of -70 mV (i) or -80 mV (ii) (the shaded areas are the standard error of the mean), **(B)** membrane time constant (tau) from -70 mV (i) and -80 mV (ii).

Once cells were held at a set prestimulus membrane potential, a series of depolarizing and hyperpolarizing current steps were injected. Injection of -40 pA of current was used to assess passive membrane properties of the cells; the averages of these traces are shown in **Figure 2**. From a prestimulus membrane potential of -70 mV the cells from the Young population charged with a mean membrane time constant of 31 ± 4 ms (*n* = 18) which was >30% faster than those from the Aged population (48 ± 4 ms, *n* = 8, Mann–Whitney U, *p* = 0.005, Z = -2.5). The membrane time constant from a prestimulus potential -80 mV averaged 29 ± 3 ms in the Young cohort (*n* = 19) and 35 ± 4 ms in the Aged cohort (*n* = 8) which was not significantly different (Mann–Whitney U, *p* = 0.14, *Z* = -1.6, **Figure 2**). There was no statistically significant effect of age on input resistance, capacitance or hyperpolarization activated “sag” (**Table 1**), although the direction of the difference in input resistance was in line with the observed membrane time constants.

**Table 1:** Passive membrane properties of Type I neurons, Young (*n* = 18), Aged (*n* = 8), statistical tests used Mann–Whitney U (MW) and repeated-measure two-way ANOVA.

	Pre-stimulus potential	Young	Aged	*P*-value	Statistical test
Input resistance (MΩ)	-70 mV	413 ± 37	539 ± 61	0.14	Repeated measure two-way ANOVA
	-80 mV	352 ± 32	420 ± 69		
Capacitance (pF)	-70 mV	79 ± 9	97 ± 15	0.16	MW
	-80 mV	90 ± 9	90 ± 8	0.6	MW
Sag (%)	-80 mV	16 ± 2	15 ± 3	0.7	MW


The spontaneous synaptic events were examined in voltage clamp mode while holding the voltage at -70 mV. The mean holding current required to maintain cells at -70 mV in voltage clamp mode was higher in the aged cohort (14 ± 4 pA, *n* = 14) than the Young group (-5 ± 5 pA, *n* = 6) (Unpaired *t*-test, *p* = 0.02, *t* = -2.5). Changes in holding voltages are often determined by two key factors, resting membrane potential and input resistance, as the input resistance of this population did not differ with age the changes in resting membrane potential are most likely to be causing the observed change. Imposed upon the baseline current of both groups was a considerable barrage of spontaneous inward-going synaptic events. The frequency of these events varied considerably from cell to cell but did not differ with age (Young: 9 ± 2 Hz, *n* = 14, aged: 10 ± 3 Hz, *n* = 6, Mann–Whitney U, *p* = 0.7, *Z* = -0.5). The average amplitude of these events was -16 pA (Young: -16 ± 1 mV, *n* = 14, Aged: -16 ± 2 pA, *n* = 6, Mann–Whitney U, *p* = 0.97, *Z* = 0.1) and was not age-dependent.

**FIGURE 3:**
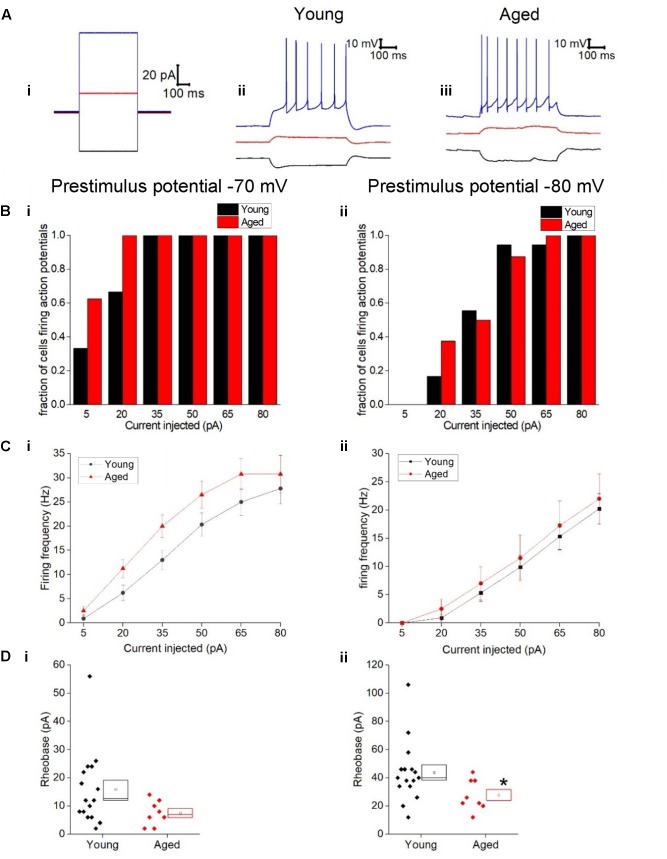
Type I neurons exhibit no age-dependent changes in excitability in response to depolarizing stimuli. **(A)** For -40 pA, +20 pA and +80 pA stimuli applied at a prestimulus voltage of -80 mV the applied current injection (i) and observed voltage response from an example Young cell (ii) and Aged cell (iii) are presented. **(B)** Fraction of cells which fired at least 1 action potential in response to each amplitude of depolarizing current injection applied from prestimulus membrane potentials of -70 mV (i) and -80 mV (ii). **(C)** Mean number of action potentials for each level of current injection from prestimulus membrane potentials of -70 mV (i) and -80 mV (ii), (error bars SEM) **(D)** Rheobase from a prestimulus membrane potential of -70 mV (i) and -80 mV (ii).

**Figure 3C** describes the mean frequency of spikes generated during the 500 ms depolarizing stimuli (including traces with zero spikes) related to stimulus amplitude, this did not differ with age (Three-way ANOVA *p* = 0.2, *F* = 1.4). Examples of current injections from both age cohorts are shown in **Figure 3**. The relationship between current injected and proportion of cells firing one or more spike is shown in **Figure 3B** for prestimulus potentials of -70 and -80 mV. As expected less current was required to generate spiking from the more depolarized prestimulus membrane potential. At one of the weaker effective current stimulus levels of 20 pA a higher proportion of cells fired in the aged cohort; from a prestimulus potential of -70 mV where 6/18 of the young cells generated action potentials while 8/8 aged cells fired (chi squared, *p* = 0.2) and from a prestimulus potential of -80 mV where 3/18 young cells fired and 3/8 aged cells fired (chi-squared *p* = 0.2), however, this failed to reach statistical significance (**Figure 3B**).

The rheobase protocol in which the increment between successive current stimuli was only 2 pA was used to examine excitability. When cells were at -80 mV significantly more current was required to produce the first spike in the Young neurons (44 ± 5 pA, *n* = 16) than cells in the Aged cohort (28 ± 4 pA, *n* = 8) (Mann–Whitney U *p* = 0.04, *Z* = -2.047, **Figure 3D**). From a prestimulus membrane potential of -70 mV the mean current required to elicit a spike was more than double in the Young cohort (16 ± 3 pA, *n* = 16) than the Aged neurons (7.5 ± 2 pA, *n* = 8) but failed to reach statistical significance (Mann–Whitney U, *p* = 0.08, *Z* = -1.8, **Figure 3D**). Finally we looked at the latency to the first action potential generated following the injection of 80 pA; no age-dependent differences were observed (-70 mV, Young: 11 ± 1 ms, *n* = 18, Aged: 11 ± 2 ms, *n* = 8, Mann–Whitney U, *p* = 1, Z = 0; -80 mV, Young: 57 ± 22 ms, *n* = 18, Aged: 85 ± 28 ms, *n* = 8, Mann–Whitney U, *p* = 0.5, *Z* = -0.7).

**FIGURE 4:**
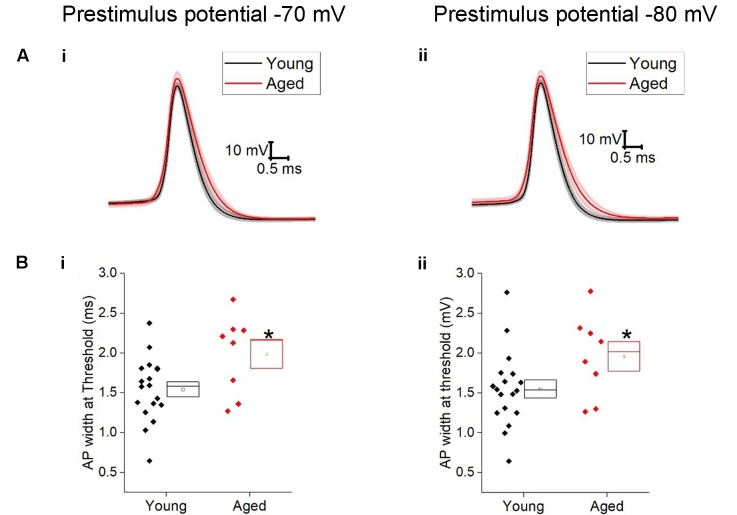
Aged Type I neurons have wider action potentials. The properties of the first action potential produced by a depolarizing pulse **(A)** averaged action potential for first Action potential from -70 mV (i) and -80 mV (ii). **(B)** action potential width at threshold from prestimulus membrane potential of -70 mV (i) and -80 mV (ii).

The action potential properties were measured from the first action potential generated in response to the series of depolarizing current injections. Average APs from the Young and Aged groups are presented in **Figure 4**. The threshold, maximum rate of rise and action potential zenith did not differ with age (**Table 2**). Spikes were noticeably broader in Aged animals, thus the action potential width measured at the threshold was a mean of >0.4 ms longer in the Aged cohort in comparison to the Young cohort (-70 mV, Young: 1.5 ± 0.1 ms, *n* = 18, Aged: 2 ± 0.2 ms, *n* = 8; -80 mV, Young: 1.6 ± 0.1 ms, *n* = 18, Aged: 2 ± 0.2 ms, *n* = 8, repeated measure one-way ANOVA, age *p* = 0.04, **Figure 4**).

**Table 2:** Action potential properties of Type I neurons Young (*n* = 18), Aged (*n* = 8), statistical tests used Mann–Whitney U (MW), repeated-measure two-way ANOVA and unpaired *t*-test.

	Pre-stimulus potential	Young	Aged	*P*-value	Statistical test
Maximum rate of rise (mV/ms)	-70 mV	269 ± 18	259 ± 27	0.6	Repeated measure two-way ANOVA
	-80 mV	297 ± 16	271 ± 26		
Threshold (mV)	-70 mV	-53 ± 1	-54 ± 2	0.9	Repeated measure two-way ANOVA
	-80 mV	-54 ± 1	-56 ± 1		
AP Zenith (mV)	-70 mV	17 ± 2	22 ± 4	0.1	Unpaired *t*-test
	-80 mV	19 ± 2	23 ± 4	0.1	MW
AP width (ms)	-70 mV	1.5 ± 0.1	2 ± 0.2	0.04	Repeated measure two-way ANOVA
	-80 mV	1.6 ± 0.1	2 ± 0.2		


### Type II Neurons

**FIGURE 5:**
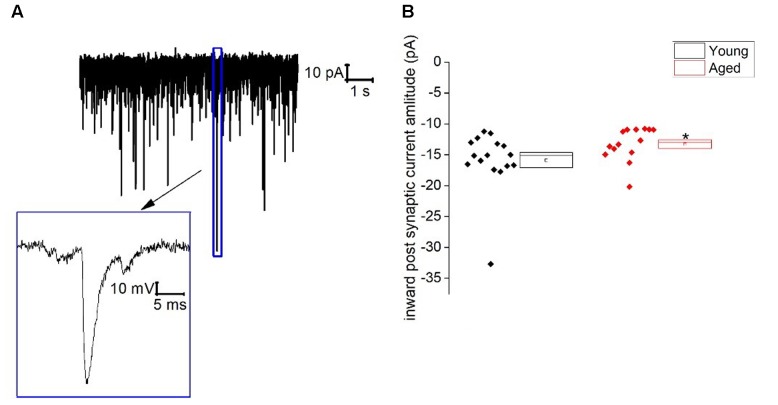
Spontaneous inward post-synaptic current properties of Type II neurons. **(A)** Sample trace from a typical BNST neuron showing numerous spontaneous inward-going postsynaptic currents and a zoomed in image of a single spontaneous synaptic events, **(B)** inward-going postsynaptic current amplitudes.

Similar to Type I neurons type II cells exhibited an ongoing barrage of spontaneous inward–going synaptic events. The frequency of these events did not differ with age (Young: 13 ± 3 Hz, *n* = 16, Aged: 7 ± 2 Hz, *n* = 14, Mann–Whitney U, *p* = 0.3, *Z* = -1.2), however, the average amplitude of these events was a mean of ∼20% larger in the Young cohort (-16 ± 1 pA, *n* = 16) in comparison to the Aged (-13 ± 1 pA, *n* = 14)(Mann–Whitney U *p* = 0.02, *Z* = -2.3, **Figure 5**). The holding current required to hold Type II cells at -70 mV in voltage clamp mode did not differ with age (Young: -6 ± 5 pA, *n* = 16, Aged: -9 ± 3 pA, *n* = 14, Mann–Whitney U, *p* = 0.5, *Z* = -0.07).

When type II cells were switched into current clamp mode there was no difference in the resting membrane potential (**Table 3**). The proportion of cells spontaneously firing, however, was far greater in the Aged cohort with 10/16 (69%) of cells firing while only 5/18 (28%) of cells fired in the Young cohort (Chi squared *p* = 0.04). The somewhat more depolarized mean resting potential is likely to be a contributing factor to the more prevalent spontaneous firing in the Aged cohort; but other factors may also contribute such as action potential threshold or input resistance. Of the cells which were spontaneously firing in the two groups there was no significant effect of age on the firing frequency (Young: 2 ± 2 Hz, *n* = 5, Aged: 4 ± 2 Hz, *n* = 10, Mann–Whitney U, *p* = 0.9, *Z* = -0.1); equally for the cells lacking spontaneous firing the rheobase was independent of age at our sample size (Young: 27 ± 4 pA, *n* = 10, Aged: 18 ± 8 pA, *n* = 5, *p* = 0.2, *t* = 1.2).

**Table 3:** Passive membrane properties of Type II neurons, Young (*n* = 18), Aged (*n* = 16) statistical tests used Mann–Whitney U (MW) and unpaired *t*-test.

	Pre-stimulus potential	Young	Aged	*P*-value	Statistical test
Resting membrane potential (mV)	NA	-73 ± 2	-69 ± 2	0.1	Unpaired *t*-test
Input resistance (MΩ)	-70 mV	482 ± 56	582 ± 69	0.3	MW
	-80 mV	433 ± 56	467 ± 68	0.8	MW
Membrane time constant (ms)	-70 mV	35 ± 4	32 ± 4	0.7	Unpaired *t*-test
	-80 mV	30 ± 4	28 ± 4	0.9	MW
Sag (%)	-80 mV	17 ± 2	21 ± 3	0.2	Unpaired *t*-test


As for type I cells the 500 ms -40 pA step of the current injection series was used to determine input resistance, membrane time constant, sag and capacitance. Input resistance, membrane time constant and sag were not age-dependent (**Table 3**). Capacitance was a mean of over 10 pF higher in the young cohort, however, this was not statistically significant with this sample size (-70 mV, Young: 74 ± 6 pF, *n* = 18, Aged: 57 ± 3 pF, *n* = 16; -80 mV, Young: 70 ± 6 pF, *n* = 18, Aged: 60 ± 3 pF, *n* = 16, repeated measure one-way ANOVA, *p* = 0.054).

**FIGURE 6:**
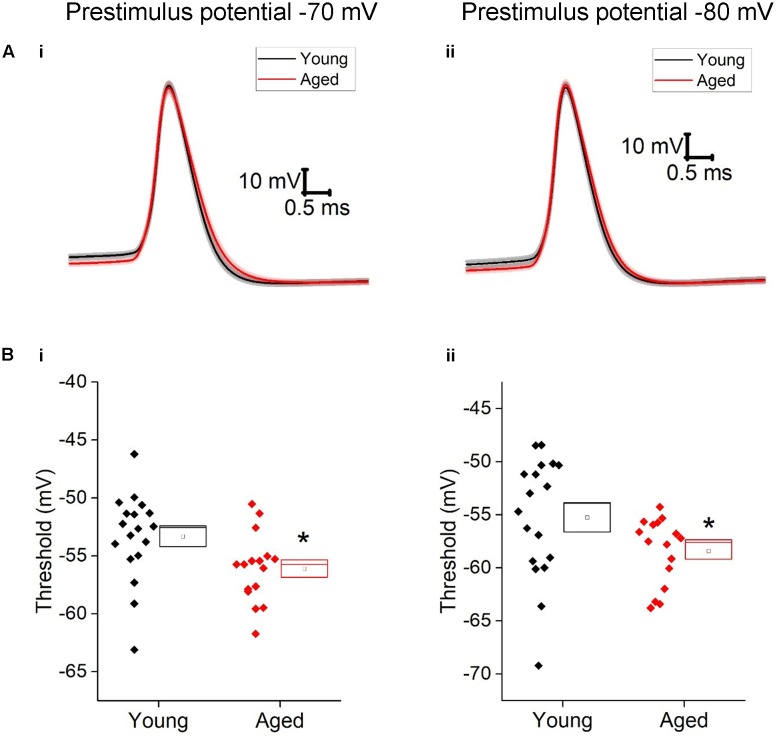
Aged Type II neurons have a more hyperpolarized action potential threshold. **(A)** averaged action potential for first Action potential from -70 mV (i) and -80 mV (ii). **(B)** Threshold from a prestimulus membrane potential of -70 mV (i) and -80 mV (ii).

The first action potential generated in response to the depolarizing 500 ms current injections was used to assess action potential properties. Threshold was more depolarized in the young cells (-70 mV, Young: -52 ± 1 mV, *n* = 18, Aged: -56 ± 1 mV, *n* = 16; -80 mV, Young: -55 ± 1 mV, *n* = 18, Aged: -58 ± 1 mV, *n* = 16, repeated measure one-way ANOVA, *p* = 0.03, *F* = 5.5, **Figure 6**). The averages for these action potentials are presented in **Figure 6**; no differences were observed in action potential zenith or the maximum rate of rise of the action potential; as the threshold differed in this population action potential width was measured from a set potential of -20 mV, this was also not dependent upon age (**Table 4**).

**Table 4:** Action potential properties of Type II neurons Young (*n* = 18), Aged (*n* = 16).

	Pre-stimulus potential	Young	Aged	*P*-value	Statistical test
Maximum rate of rise (mV/ms)	-70 mV	260 ± 18	254 ± 20	0.12	Repeated measure two-way ANOVA
	-80 mV	283 ± 19	290 ± 17		
AP width (ms)	-70 mV	0.8 ± 0.02	0.8 ± 0.03	0.14	Repeated measure two-way ANOVA
	-80 mV	0.7 ± 0.03	0.8 ± 0.02		
AP Zenith (mV)	-70 mV	18 ± 2	17 ± 2	0.97	Repeated measure two-way ANOVA
	-80 mV	17 ± 2	18 ± 2		


The shift in action potential threshold appeared to have no effect on the amount of current required to produce at least one spike in the “rheobase” protocol both set prestimulus membrane potentials (-70 mV, Young: 14 ± 2 pA, *n* = 16, Aged: 15 ± 5 pA, *n* = 13, Mann–Whitney U, *p* = 0.3, *Z* = -1; -80 mV, Young: 35 ± 3 pA, *n* = 15, Aged: 34 ± 8 pA, *n* = 14. Mann–Whitney U *p* = 0.4, *Z* = -0.9, **Table 4**).

**FIGURE 7:**
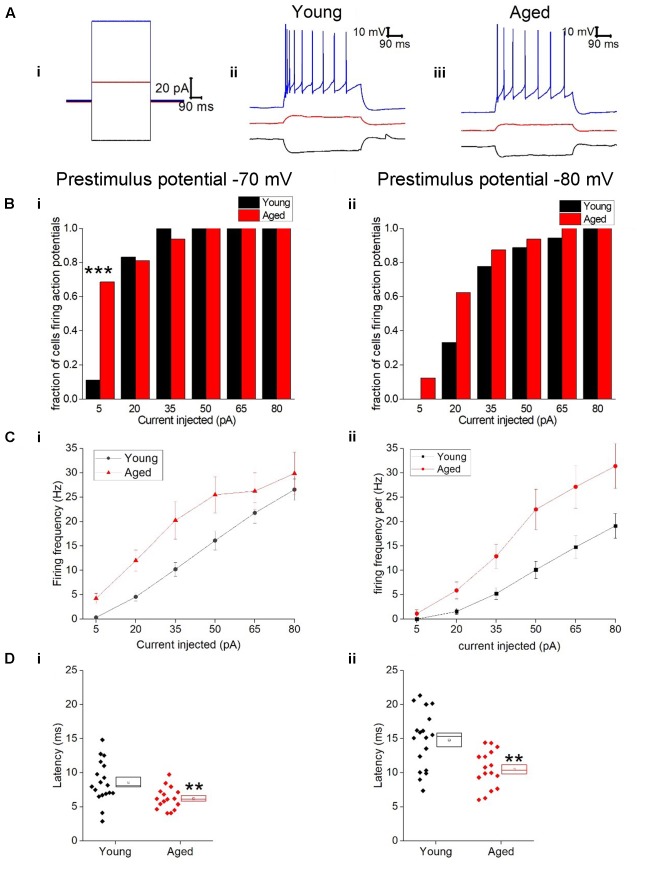
Type II neurons exhibit hyperexcitability in aged neurons in response to depolarizing stimuli. **(A)** For -40 pA, +20 pA and +80 pA stimuli applied at a prestimulus voltage of -80 mV the applied current injection (i) and observed voltage response from an example Young cell (ii) and Aged cell (iii) are presented. **(B)** Fraction of cells which fired at least 1 action potential in response to each amplitude of depolarizing current injection applied from prestimulus membrane potentials of -70 mV (i) and -80 mV (ii), significance based on chi Squared tests. **(C)** Mean number of action potentials for each level of current injection from prestimulus membrane potentials of -70 mV (i) and -80 mV (ii), respectively (error bars SEM), **(D)** Latency from a prestimulus membrane potential of -70 mV (i) and -80 mV (ii).

Given the more hyperpolarized threshold of type II neurons in aged animals one might expect to see an increase in excitability in these cells. Excitability was examined using a series of incremental 500 ms depolarizing current injections. Sample traces containing components of these protocols can be seen in **Figure 7**; firstly we examined whether or not the cell fired in response to injections of depolarizing currents (**Figure 7**). From a prestimulus membrane potential of -70 mV the first step (5 pA) caused a higher proportion of Aged cells to fire one or more action potentials with 11/16 (69%) Aged cells firing while only 2/19 (11%) cell from Young animals produce a spike (chi-squared *p* = 0.0004). There was no difference in the likelihood of firing in any other steps from this prestimulus membrane potential. This is most likely due to the fact that at the 20 pA injection >78% of cells in both populations generated at least 1 spike, and at 50 pA step all cells in both cohorts were firing. From -80 mV the likelihood of firing did not differ for any of the current steps. Another measure of excitability is the average frequency of action potentials during each of the current steps. There were significantly higher rates of firing in the Aged cohort in comparison to the Young (Three way ANOVA *p* = 0.015, *F* = 6.7, **Figure 7C**). Furthermore, following the injection of 80 pA of depolarizing current the Aged neurons generated spikes ∼33% earlier their Younger counterparts (-70 mV, Young: 9 ± 0.7 ms, Aged: 6 ± 0.4 ms; -80 mV, Young: 15 ± 1 ms, Aged: 10 ± 1 ms, repeated measure one-way ANOVA, *p* = 0.002, *F* = 12).

## Discussion

The data presented here describe differences in the intrinsic properties of two classes of BNST neurons observed when comparisons were made between patch clamp recordings performed in the anterolateral area of the BNST in brain slices obtained either from post-pubescent Young (3–4 months) or very Aged (29–30 months) female C57-BL6 mice. In work by others, neurons in this area have been classified into three types (Hammack et al., 2007; Daniel and Rainnie, 2016; Daniel et al., 2017). Due to the low numbers of neurons with a type III phenotype their physiological role in aging was not addressed here. Overall our data point to intrinsic hyper-excitability associated with normal aging in type II neurons and a substantial hyperpolarisation of the resting membrane of Type I cells with aging.

In type I neurons the mean resting membrane potential of the Aged neurons was >10 mV more hyperpolarized. This would place the cells away from action potential threshold, which was similar at the two ages, making them less likely to fire, however, there was no significant difference in the proportion of Type I cell firing at rest. The differences in prestimulus membrane current for both the voltage clamp recordings and the current clamp recordings performed at set potentials can be accounted for by these differences in resting membrane potential, in the face of relatively similar input resistances. In Type II neurons a significantly higher proportion of the Aged cohort were firing at rest.

The spontaneous action potential firing seen in these neuronal populations of the BNST neurons suggest this area may be able to provide a substantial tonic drive to their various downstream synaptic targets, which included the CRF releasing cells of the PVN as well as the CRF circuit within the BNST (Daniel and Rainnie, 2016). This does assume, however, that the intrinsic properties seen in acute brain slices are truly reflective of those present *in vivo*. This commonly made assumption is one we have certainly questioned previously in the light of how intrinsic properties can be readily and persistently modified by “neurophysiological experiences” (e.g., periods of tonic depolarization) that might be expected to occur during the process of preparing acute brain slices (Brown and Randall, 2009). It should also be appreciated that the use of whole cell recording will make the cytoplasmic contents of the young and old cells identical, and consequently any differences in intracellular ion concentrations that are age-associated will be lost along with their neurophysiological consequences.

In voltage-clamped type II neurons, the average inward-going spontaneous synaptic event was, on average, ∼2.5 pA larger in the Young cohort. In other unpublished work we have shown that these synaptic events are blocked by addition of glutamatergic blockers in post-pubescent C57-BL6 females when using the same solutions as described here. A higher average amplitude of synaptic events could increase probability of firing, however, this was not observed once the cells were switched into current clamp most likely due to differences in the intrinsic properties which lead to Aged neurons being hyper excitable.

The use of brain slices will also impact on the “functional” connectivity of the BNST. Thus, although the excitatory and presumably inhibitory synapses onto BNST neurons remain [as evidenced by the spontaneous synaptic activity (**Figure 5**)], their afferent axons are for the most part detached from their cell-bodies, and thus are highly likely to be silent. Consequently, one must also consider the level of spontaneous neural activity in the light of this functional denervation. For example, the preparation of slices and the use of a high chloride intracellular solution could have disrupted some form of strong inhibitory synaptic tone that *in vivo* holds BNST cells more negative reducing electrogenesis of spikes. Having said this, some of the very first neurophysiological studies of the BNST, *in vivo* recordings in anesthetized female rats, revealed that the majority of BNST neurons that exhibited any activity fired at >1 Hz, and this activity may have to some degree been affected by estrogen status (Bueno and Pfaff, 1976). More recently *in vivo* recordings from ventral BNST in awake male mice indicate that, although heterogeneous, firing rates in active cells that project to VTA average around 5 Hz, similar to the spontaneous firing level we see in slices (Jennings et al., 2013).

When the membrane potential was pre-set to fixed levels of either -80 or -70 mV prior to application of current stimuli, the depolarization-evoked action-potential frequency was higher in type II Aged animals. Theoretically such age-associated increase in excitability could result from a number of underlying sources. These include, but are not limited to, a negative shift in AP threshold (akin to that we reported in hippocampal neurons, Randall et al., 2012), changes to the kinetics of Na^+^ channel repriming, altered AHPs, or differences in membrane conductances active near threshold, that either promote (e.g., low threshold Ca^2+^ channels) or resist (low threshold K^+^ channels) AP genesis. Furthermore, quite subtle changes in combinations of these factors could synergize to promote enhanced excitability. In the aged cohort a significant hyperpolarized shift in action potential threshold of Type II neurons were seen. Using macropatch recordings from hippocampal neurons we have previously shown how aging-associated changes in action potential threshold were paralleled by equivalent shifts in the voltage-dependence of Na^+^ channel gating (Randall et al., 2012), although in that cellular population action potential threshold was more depolarized in Aged animals, the opposite to our findings here. In future it would be interesting to take a similar approach to examine the shift in AP threshold in BNST neurons. Interestingly the magnitude of the shift may be greater when cells are more depolarized, which may reflect age-associated shifts in the inactivation curve of Na^+^ channels underlying spike initiation. The differences in threshold of Type II neurons could account for the higher proportion of cells spontaneously firing at rest.

The majority of neurons in the BNST are GABAergic; BNST originating GABAergic projections innervate multiple areas of the CNS, for example those involved in reward, feeding and stress (Dong et al., 2001; Dong and Swanson, 2004; Forray and Gysling, 2004). One key pathway is that monosynaptically linking BNST neurons to CRF producing cells of the PVN (Choi et al., 2007). It is likely that this is the predominant pathway in mediating reductions in plasma glucocorticoid levels in response to BNST stimulation. Furthermore, BNST cells that are activated by excitatory inputs from limbic structures such as the medial prefrontal cortex and hippocampal formation can, in turn, mediate a direct inhibitory, GABAergic drive to CRF-releasing neurons in the PVN (Cullinan et al., 1993, 2008; Boudaba et al., 1996). Lesion of this system leads to exaggerated HPA activation by stressors (Radley and Sawchenko, 2011) suggesting that the mPFC inputs to the BNST can act as a brake on activity in the HPA axis. Afferent inputs to the BNST from the central amygdala, in contrast, are inhibitory and thus rely on the influences of the BNST (and consequently afferent inputs from mPFC) on PVN activity.

An interesting observation seen in Type I neurons was a ∼25% widening of action potentials with age. If this outcome, seen in our recordings at the cell body, translated to similar changes in spike width at the presynaptic terminals formed by BNST neurons, a substantial increase in release probability might be expected, and thus a corresponding increase in drive to down-stream synaptic targets of BNST neurons. This might be expected to increase the inhibitory influence of BNST on the PVN and thus reduce overall HPA activity by limiting CRF output from PVN neurons (Seeman et al., 2001; Dalm et al., 2005).

This increase in excitability in the type II neurons could also contribute to a decrease in anxiety with age. This study did not address the anxiety levels of the mice prior to culling, however, in humans a decrease in the prevalence of anxiety disorders with age has been reported (Byers et al., 2014). As this cell type is thought to act as an ‘anxiety off’ switch (Daniel and Rainnie, 2016) its increased excitability could lead to a greater GABAergic inhibition of the CRF cells both in the PVN and within the BNST itself.

Although it can be proposed that neurophysiological changes in BNST neurons develop as a consequence of what might be regarded as autonomous aging-related processes within the BNST itself, another possibility is that the aging-associated neurophysiological changes that arise in type I and type II cells represents an adaptive change in response to an altered afferent synaptic drive from, for example, other limbic structures. Certainly other components of the limbic system with well-established BNST connectivity have been previously shown to exhibit significant neurophysiological changes associated with aging (Chang et al., 2005; Bodhinathan et al., 2010; Coskren et al., 2014).

The BNST is well known for its sexually dimorphic nature; due to their availability for this first study of BNST neurophysiology in Aged animals we focused entirely on females. An interesting extension to this work would be to compare how aging alters the neurophysiological properties of male mice. It would also be of interest to investigate how neurophysiological profiles in sub-regions of the BNST respond to aging, although such work would perhaps be better performed in rats. This is because subregions have been better defined in that species and also the greater physical size of the rat brain would make such studies more experimentally tractable. Notably our data provide no indication of when the neurophysiological changes we report develop within the mouse lifespan. A more detailed longitudinal study would be required for this, preferably sampling multiple age points from a large cohort of littermate animals raised throughout life in the same facility.

Overall this study identified a hyper-excitability of Type II neurons and a hyperpolarized resting membrane potential of Type I in aged female mice. Due to the central role of the anterolateral BNST in the limbic influences over the HPA axis, the changes observed in this study could shape changes of HPA axis function with age.

## Author Contributions

HS planned and carried out the experiments, she was also responsible for the analysis of the data and contributed significantly to the writing of the manuscript. JT was involved in the securing of the initial grant and also proved expertise in stress pathways. JB was involved in the analysis of the data, provided all the Matlab scripts used during analysis and contributed to the statistics used. AR was involved in the securing of funding, the analysis of the data and the preparation of the manuscript.

## Conflict of Interest Statement

The authors declare that the research was conducted in the absence of any commercial or financial relationships that could be construed as a potential conflict of interest.
